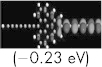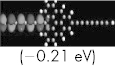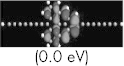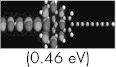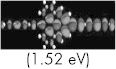# All-carbon sp-sp^2^ hybrid structures: Geometrical properties, current rectification, and current amplification

**DOI:** 10.1038/srep02575

**Published:** 2013-09-03

**Authors:** Zhenhua Zhang, Junjun Zhang, Gordon Kwong, Ji Li, Zhiqiang Fan, Xiaoqing Deng, Guiping Tang

**Affiliations:** 1 School of Physics and Electronic Science, Changsha University of Science and Technology, Changsha 410114, China; 2 College of Natural Science, the University of Texas at Austin, Austin, Texas 78712, USA

## Abstract

All-carbon sp-sp^2^ hybrid structures comprised of a zigzag-edged trigonal graphene (ZTG)and carbon chains are proposed and constructed as nanojunctions. It has been found that such simple hybrid structures possess very intriguing propertiesapp:addword:intriguing. The high-performance rectifying behaviors similar to macroscopic p-n junction diodes, such as a nearly linear positive-bias *I-V* curve (metallic behavior), a very small leakage current under negative bias (insulating behavior), a rather low threshold voltage, and a large bias region contributed to a rectification, can be predicted. And also, a transistor can be built by such a hybrid structure, which can show an extremely high current amplification. This is because a sp-hybrid carbon chain has a special electronic structure which can limit the electronic resonant tunneling of the ZTG to a unique and favorable situation. These results suggest that these hybrid structures might promise importantly potential applications for developing nano-scale integrated circuits.

The motivation for the miniaturization of electronic devices has driven the intensive electronics research activities into low-dimensional material systems, specially focusing on carbon-based nanostructures^1^,^2^. As one of the most promising candidates, two-dimensional graphene^3^ provides a new opportunity for the carbon-based nanoelectronics^3^,^4^. In particular, graphene nanoribbons (GNRs)^5^,^6^,^7^,^8^ have attracted much attention due to a rich variety of band gaps, from semimetals to wide-gap semiconductors. Another class of important graphene derivatives is finite-size 0D graphene nanoflakes (GNFs) with triangular, rectangular and hexagonal shapes^9^,^10^,^11^,^12^,^13^. They are nanometer-scale materials which have closed edges. GNFs can be constructed by connecting several benzenes, some of which have already been manufactured by soft-landing mass spectrometry^14^,^15^. However, currently, the excellent experimental method to obtain various GNFs is by cutting a graphene along specific single crystallographic directions^16^. The quantum confinement and finite-size effects for GNFs would influence their electronic properties greatly, which has been studied extensively^9^,^10^,^11^,^12^,^13^. Especially, the experiments have achieved GNFs features at the scale of tens of nanometers^17^. Among GNFs, zigzag-edge trigonal graphenes (ZTGs) are prominent in their electronic property because there exist half-filled zero-energy states. This novel property was revealed by both tight-binding model^11^ and first-principles calculation^18^,^19^.

Linear carbon chains Cn are also a kind of important carbon nanostructures made of *sp*-hybridized C atoms. Their special structure and electronic properties are attracting more and more investigation interest both theoretically^20^,^21^,^22^,^23^ and experimentally^24^,^25^,^26^. Carbon chains are traditionally classified as cumulene (monatomic chains with double bonds, = C = C = ) or polyyne (dimerized chains with alternating single and triple bonds, −C≡C−),and one of their distinct electronic properties is the odd-even property of conductance^20^,^21^,^22^,^23^. The classical and first-principles molecular dynamics simulations showed that long linear atomic chains could be pulled from graphene^27^ or from mechanically stretched graphene-based materials^28^. Recently, two research groups have successfully carved out carbon chains from graphene by using a high-energy electron beam^29^,^30^,^31^. Thus, linear atomic carbon chains can also be regarded as a family of graphene derivatives or extremely narrow GNRs, which can be used as a transport channel as on-chip interconnects for molecular electronic nanodevices. This choice could bypass the two challenges^22^: one is the difficulty in getting sub-10-nm width semiconducting GNRs due to the limitation of the current lithography technique, and another is chirality. As we known, it is hard to control appropriate chirality in the process of fabricating GNRs, which would affect to meet special transport requirements of graphene-based devices, while atomic carbon chains are atomically thin and not chiral. Nevertheless, up to now, a linear carbon chain is only viewed as a single molecule for studies, and effects of its modification on other nanostructures is still largely unexplored.

In this paper, we propose that using two kinds of graphene derivatives, ZTG and carbon chains C_n_, constructs the all-carbon hybrid structure: carbon chain/ZTG/carbon chain. The basic purpose is to apply carbon chains acting as the conductive channel for the ZTG, more importantly, to modify the geometrical structure and electronic features of the ZTG by carbon chains in attempt to create unexpected effects. Our calculations from the first-principles method show that electronic structures and transport of this hybrid structure remarkably demonstrate even-odd property related to the number of carbon atoms on the chain, especially for its rectifying direction and strength. Interestingly, the high-performance rectifying behaviors similar to a traditional diode and the transistor property for an extremely high current amplification can be predicted by such hybrid structure.

## Results

**Figure 1:**
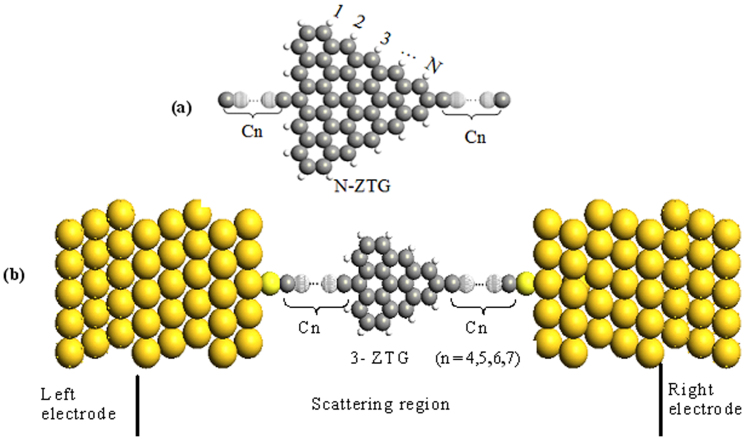
Geometrical structure. (a) The general hybrid structure of Cn/N-ZTG/Cn. (b) Nanojunctions comprise of Cn/3-ZTG/Cn (n = 4,5,6,7) contacted with Au electrodes.

Figure 1(a) shows the general hybrid structure of carbon chain/ZTG/carbon chain. Here, we only consider the case of the ZTG having equilateral sides and linked with two symmetric equi-length carbon chains. The ZTGs with various sizes is defined as N-ZTG, where N denotes the number of edge hexagonal cells in one side of the triangle, and its zigzag edges are terminated by H atoms to eliminate the dangling bonds on edge carbon atoms. The length of a carbon chain at each side of the ZTG is referred to as Cn, where n denotes the number of carbon atoms in the chain. To investigate electronics features of such a hybrid structure, each optimized structure by a separate calculation based on DFT is positioned between two (5 × 5)Au (111) electrodes through S atoms and is initially chosen to sit upright on the Au surface with a typical Au–S distance of 2.45 Å to construct a nanojunction, as shown in Figure 1(b), where S atoms, acting as “anchoring atoms”, form strong Au–S bonds with Au atoms and make this hybrid structure capable of a chemical adsorption on the Au surface, therefore, a very small resistance occurs at their interface (ohmic contact). In order to avoid an overmuch calculating cost, we only select one very small TGN, 3-TGN, as a representative. For its attached carbon chains Cn, we choose n = 4, 5, 6, and 7 to evaluate the length effects of carbon chains, and corresponding nanojunctions are referred to as M1, M2, M3, and M4, respectively.

**Figure 2:**
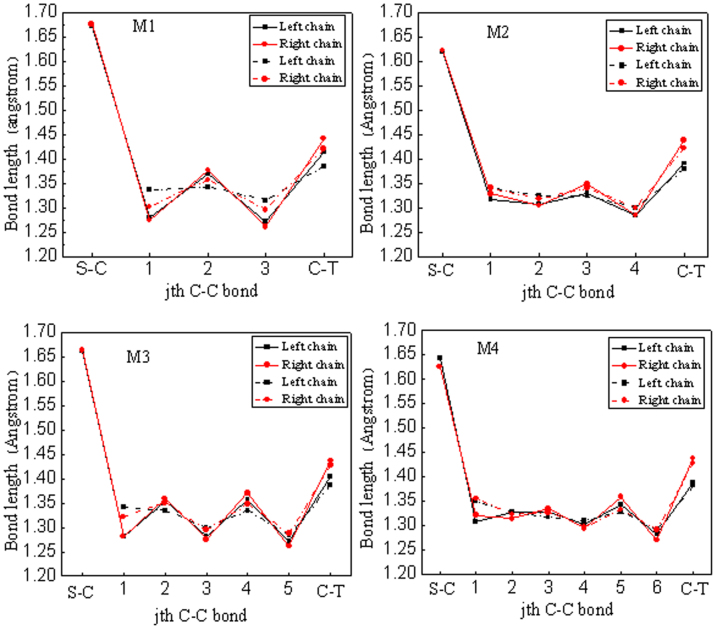
The bond-length properties of the chain. All dotted lines and solid lines indicate the bond length of a chain for a pure hybrid structure and nanojunction, respectively. S-C is the bond length between S atom and C atom at one end of a carbon chain, and C-T denotes the bond length of the C atom at another end of a carbon chain connecting the ZTG.

The bond-length properties for the carbon chain are shown in Figure 2,where S-C denotes the bond length between S atom and C atom at one end of a carbon chain, and C-T is the bond length of C atom at another end of a carbon chain connecting the ZTG. All dotted lines in figures indicate the bond length of chain for pure hybrid structures. Obviously, the C-T is longer than the C-C bonds in the carbon chain. This is because the carbon chain is terminated at the sp^2^ carbon atom which is bonded to adjacent two carbon atoms in the ZTG with triple bonds, and in turn a longer single bond occurs for the C-T. The C-C bonds in the carbon chain exhibit a cumulene-like configuration but with a non-negligible bond-length alternation (BLA). The similar result that the carbon chain forms a stable cumulene-type structure when it is bonded to the sp^2^-kind termination has been observed in other studies^21^. After above-stated hybrid structures are constructed as nanojunctions, the bond-length properties for the chain are displayed by solid lines, as shown in Figure 2 as well. As can been seen, the situation is altered greatly. C-C bonds in the carbon chain trend to become polyyne-like configuration from cumulene-like one, especially prominent for M1 and M3, which have even number of carbon atoms on the chain. This alteration can be attributed to the bonding nature of S atom. The very strong Au-S bond tries to raise the energy of a system, however, in order to keep the stability of system, a polyyne-like configuration is produced to lower the energy, like a Peierls-type distortion, where the structure with the single and triple bond alternation has a lower energy. Additionally, our study implies that the traditional categories of strict polyynes with a large BLA and strict cumulenes with a negligible BLA are too simple to describe our system. On the other hand, the C-H bond length is 1.16–1.17 Å, and C-C bond length in the ZTG appears in a range of 1.40–1.45 Å with larger values internally than those externally.

In order to give a visual description for the electronic structure, we first calculate the zero-bias molecular projected self-consistent Hamiltonian (MPSH) eigenstates for all nanojunctions, as shown in Table 1, where the Fermi level *E_f_* is set as zero. The MPSH is the self-consistent Hamiltonian of the molecule in the presence of the Au electrodes, and thus it contains the molecule–electrode coupling effects during the self-consistent iteration. For the spatial distribution of molecular states, two important features are visible: (1) except that the highly located HOMO at the Fermi level originates only from the molecular state of the ZTG, other orbitals all derives from carbon chain states or state hybridizations of carbon chains to the ZTG. This means that the carbon chain has a strong tuning ability on electronic structures of the whole hybrid structure, and (2) they demonstrate an obvious odd-even properties, namely, their electronic structures are strongly dependent on the number of carbon atoms in a chain being odd or even, not only being reflected by level positions of orbitals, but also by the spatial distribution of molecular states, particularly obvious for the LUMO and HOMO + 1. These will unambiguously result in odd-even properties for the transport of nanojunctions. Additionally, we can see that the HOMO for all nanojunctions and the HOMO-1 for M2 and M4 are highly localized states, while LUMO + 1 for all nanojunctions and LUMO for M1 and M3 are highly localized states, and the delocalized degree of other orbitals is medium. It is worth noting that these molecular orbital does not make an equal contribution to the electron transmission, a large contribution comes from a delocalized orbital, and less or no contribution is made by a localized one because its tunneling ability is suppressed.

**Figure 3:**
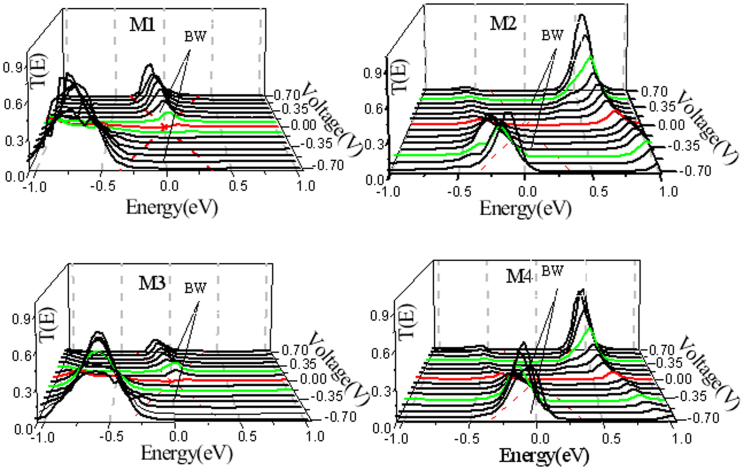
Transmission spectra of all models in the bias region from −0.7 V to 0.7 V. The middle region of both red dotted lines denoted the bias window (BW).

The transmission coefficient *T*(*E*,*V*) as a function of the energy level *E* at a certain bias *V* can be calculated by using the formula, *T*(*E*,*V*) = *T_r_*[Γ*_L_*(*E*)*G^R^*(*E*)]Γ*_R_*(*E*)*G^A^*(*E*)], where *G^R^*(*E*) and *G^A^*(*E*), respectively, are the advanced and retarded Green's functions of the scattering region, and 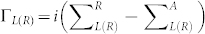 is the coupling functions of the conductor to the left and right electrodes, 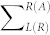 is a self-energy matrice used to include effects of the left (right) semi-infinite electrode, which is explicitly include in the Kohn-Sham calculation for the extended molecule system. Our calculated transmission spectra for all models in the bias region from −0.7 to 0.7 V in steps of 0.1 V is displayed in Figure 3, where the region of voltage [−*V*/2, +*V*/2] is the bias window for the electronic transport, which is denoted by two red dotted lines. The distinctive features for transmission spectra are their high asymmetry in the bias window with respect to different polarities of biases, thus a strong rectifying behaviors can be expected. More interestingly,their odd-even property related to a carbon chain is able to be clearly seen as well. For M1 and M3 with the even number of carbon atoms in a chain, the transmission peak always stays inside the bias window (BW) under positive bias and keeps outside the BW under negative bias. In contrast, for M2 and M4 with the odd number of carbon atoms in a chain, the transmission peak runs into the BW under negative bias and almost moves outside the BW under positive bias. These distinctions therefore predict an opposite rectification direction for M1 (M3) and M2 (M4).

**Figure 4:**
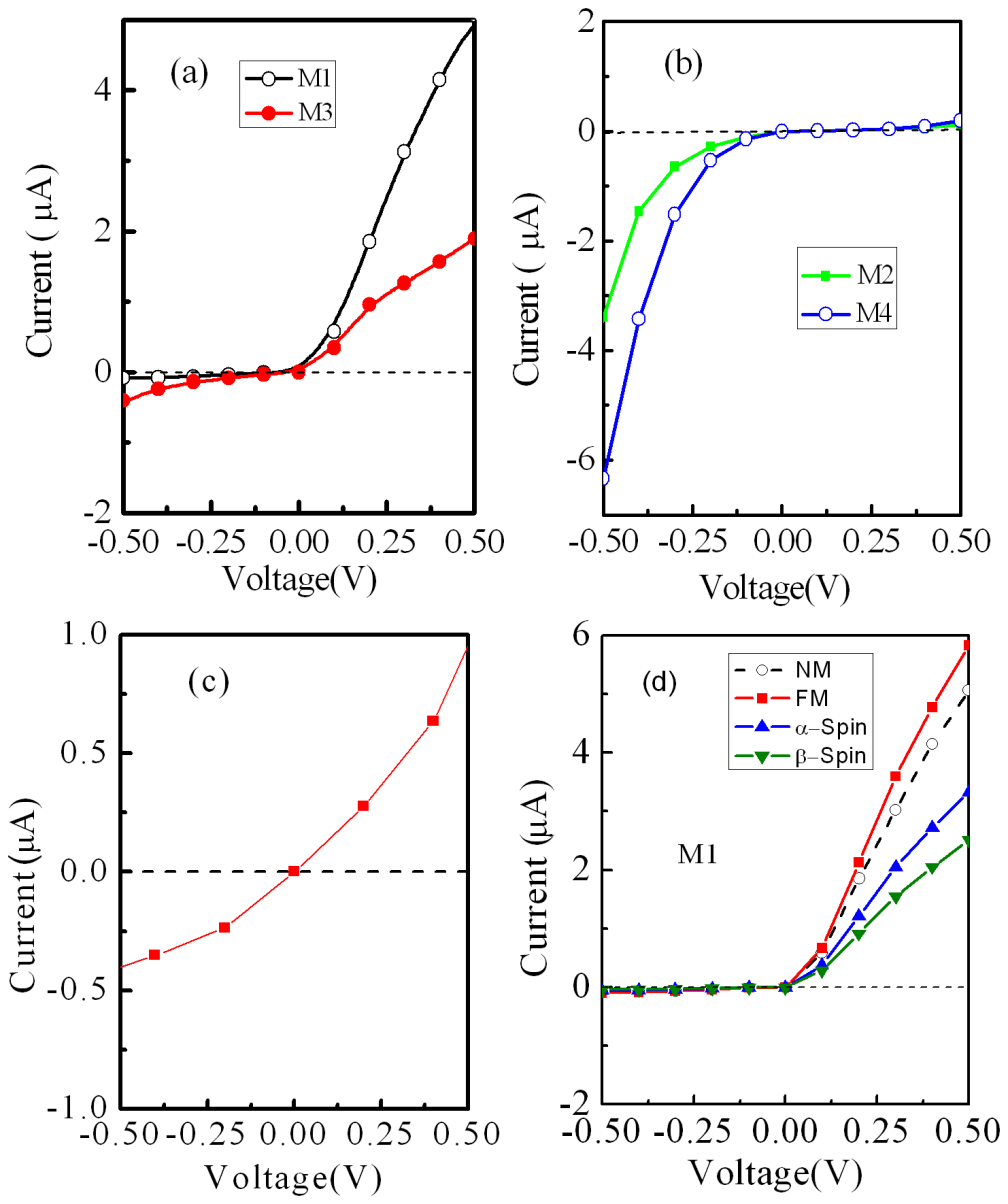
The I-V characteristics. (a) and (b) The I-V characteristics for all nanojunctions we construct. (c) The I-V characteristics for a nanojunction constructed by sandwiching a single 3-ZTG (without carbon chains on its two sides) between two Au electrodes. (d) The spin-polarized I-V characteristics. NM and FM indicate the non-magnetic state and the ferromagnetic state (α-spin plus β-spin), respectively.

The current *I* through a nanojunction can be calculated from the Landauer-like formula^32^: 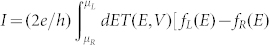, where *μ_R_* (*μ_L_*) is the chemical potential of the right (left) electrode and the difference of them is *μ_L_* − *μ_R_* = *eV*, and *f_L,R_* are the Fermi function for left and right electrodes. The self-consistently calculated current- voltage (*I-V*) characteristics for all nanojunctions in the bias range from −0.5 V to 0.5 V are shown in Figure 4 (a) and (b). We can see that their I-V curves display highly asymmetric behaviors. M1 and M3 have a much larger current under positive bias than that under negative bias, while M2 and M4 are in quite contrast, a larger current happens under negative bias. These observed results agree well with the calculated transmission spectra, as shown in Figure 3. These highly asymmetrical *I-V* characteristics suggest that such hybrid structures have a strong current rectification. Apparently, nanojuctions with an even number of carbon chains, M1 and M3, have a forward rectification, but the rectification behavior is weakened with increasing the length of a carbon chain. While for nanojuctions with an odd number of carbon chains, M2 and M4, the opposite situation appears, they have a reverse rectification, and the rectification can be enhanced by increasing the length of a carbon chain. As a comparison, we also calculate the I-V characteristics for a nanojunction constructed by sandwiching a single 3-ZTG (without any carbon chains on its sides) between two Au electrodes, as shown in Figure 4(c). One can see that almost no rectification can be achieved. Therefore, such hybrid structures possess two very intriguing features: One is that carbon chains have an extremely strong modulating ability for transmission and transport properties and make its rectification behaviors be promoted greatly. Another is that only altering the number of carbon atoms in a chain by one (the odd-even alteration of the carbon atom number) can obviously lead to a change of the rectification direction. These may have unique applications for developing a new type of nanodevices. More importantly, for M1, the highly asymmetrical *I-V* characteristic is similar to that of the traditional diode, which has a nearly linear positive-bias *I-V* curve (metallic behavior), a very small leakage current under negative bias (insulating behavior), a rather low threshold voltage (<0.1 V) to operate such a device, and a large bias region for a rectification, thus M1 is more suitable to act as a high performance nano-diode as compared with other nanojunctions. Additionally, we have also calculated the *I-V* characteristics of the ZTG linked with longer carbon chains Cn, such as n = 8 and 9, and find that their rectification direction follows the same odd-even property as the models M1–M4, but their leakage currents increase, that is, the performance of their rectification is weakened. This is easily understood because a longer carbon chain would cause a decline of the geometrical asymmetry of the hybrid structure.

As we know, the ZTG has an intrinsic spin polarization. In order to test such an effect on the I-V characteristics, we take M1 as an example and the spin-polarized density function theory. The spin-polarized I-V characteristics are shown in Figure 4(d). As a comparison, the I-V characteristics for the non-magnetic state (NM), which has been shown in Figure 4 (a), and the total I-V characteristics (α-spin plus β-spin) for the ferromagnetic state (FA) are also plotted in Figure 4(d). We find that the I-V characteristics for NM and FA states have only minor quantitative differences. However, the previous works demonstrated that the spin-polarized state would become unstable with respect to the spin-unpolarized state at finite temperature^33^ or in the presence of a ballistic current through the GNRs^34^. Therefore, it might be most possible experimentally detectable electronic transport behaviors for the ZTG with the NM state.

**Figure 5:**
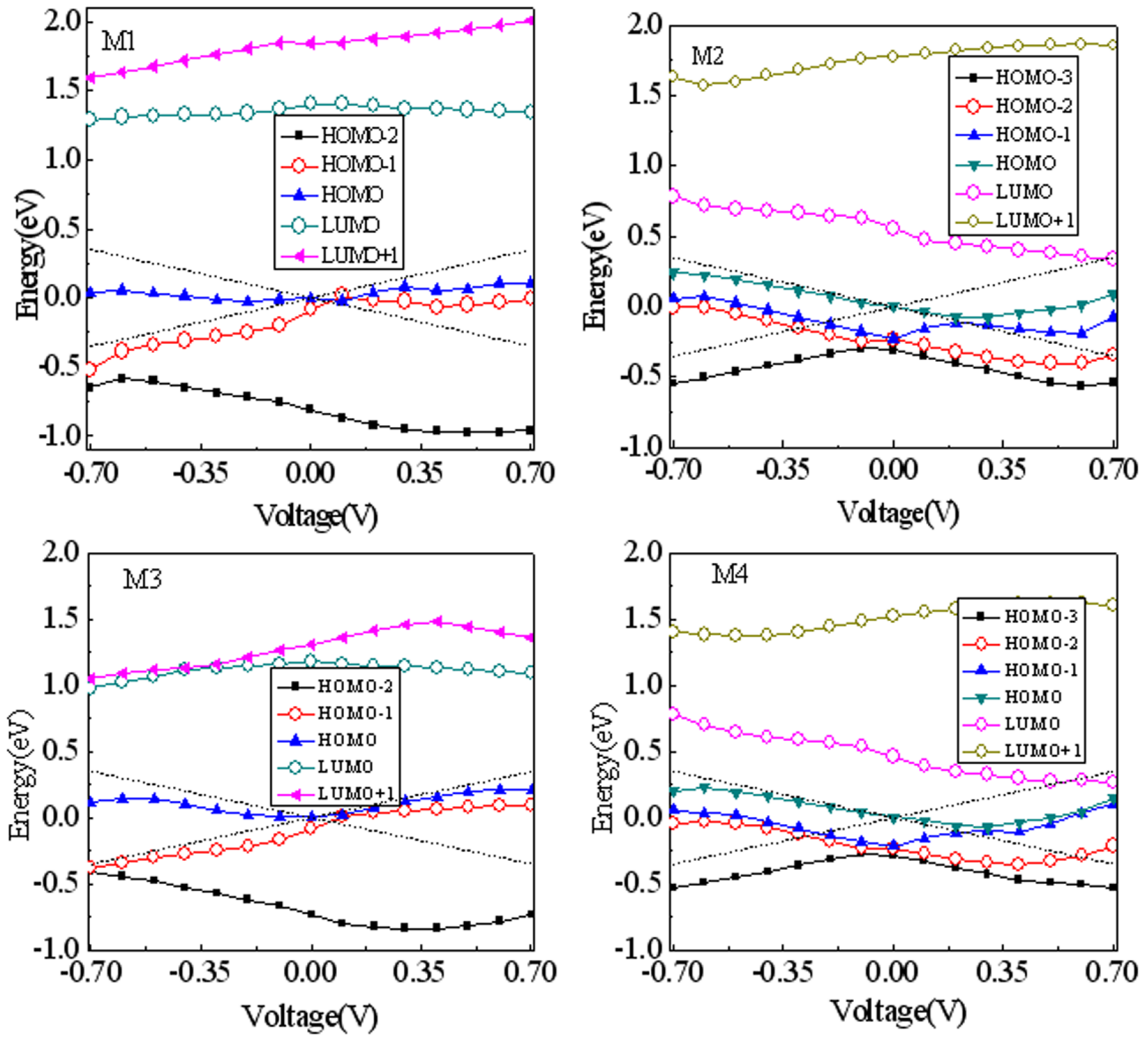
Evolution of the molecular orbital (MO) levels with applied bias for all nanojunctions. The MO denoted by lines with open circle symbols features a relative higher delocalization and makes a large contribution to the electronic tunneling.

To address the underlying origins for transmission and transport properties, particularly for rectifying behaviors, we give an evolution of the molecular orbital (MO) levels with applied bias for all nanojunctions, as shown in Figure 5. One can observe that the asymmetric shift of the MO under positive and negative biases, which serves to a rectification of the nanojunction. In particular, the MOs denoted by lines with open circle symbols feature a relative higher delocalization, which are LUMO and HOMO-1 for M1 and M3, and LUMO and HOMO-2 for M2 and M4. These MOs make a large contribution to the electronic tunneling through a molecular core and thus basically determine transport properties, including the rectification strength and direction. For M1 and M3, the HOMO-1 always stays inside the BW under positive bias and keeps outside the BW under negative bias, thus a forward rectification occurs. But for M2 and M3, the HOMO-2 runs into the BW under a lower negative bias, thus a reverse rectification appears. Additionally, the large transmission peaks at a transmission spectrum curve observed in Figure 3 correspond to these relative higher delocalized OMs or a combination with other OMs. While for those highly localized MOs, their tunneling ability is strongly suppressed so that no or small transmission peaks pop up in the transmission spectrum curves. Particularly for M1, we can see that an appearance of the low threshold voltage is because the delocalized HOMO-1 enters into the positive-bias window at a very low bias(<0.1 V), and a very small leakage current originates from a localized HOMO located entirely on the ZTG, as shown in Table 1. In addition, we can observe that different evolution behaviors for different orbitals with bias. This is intimately related to the localized position of a molecular state. If a molecular state is localized on the left (right) side of the molecule, which implies that this state has a strong coupling with the left (right) electrode so that this orbital level always follows the left (right) electrode under bias^35^,^36^,^37^, the orbital level then moves up (down) with bias to form a straight line. However, the state localized at the one side might be sensitive to bias, this will lead to a change of the localized position of the molecular state with bias and thus to form a polygonal line or an irregular line for the shifting orbital level.

**Figure 6:**
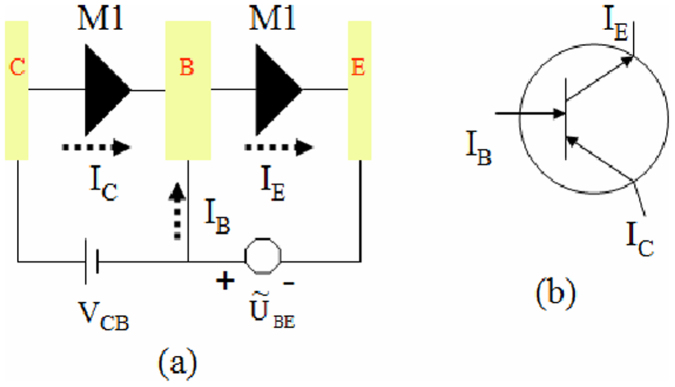
The current amplification circuit. (a) Structure of the transistor composed of an integration of two M1. (c) The circuit diagram and current direction of the transistor.

**Figure 7:**
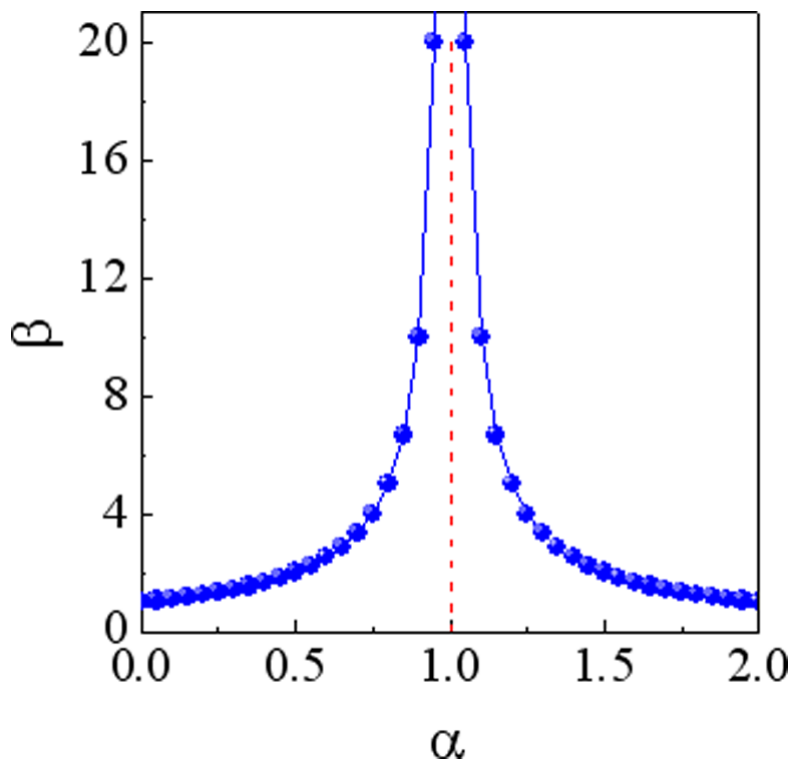
The β-α relation. When α = 0 or 2.0, β = 1.0 (no amplification effect). But the β is enhanced dramatically when the α value approaches 1.0, corresponding to a considerable current amplification.

As stated above, M1 can serve as a particular diode with a very low threshold voltage, a very small leakage current, and a wide bias region for rectification. Here, we show that it is possible to integrate two M1 for constructing a three-terminal device, transistor, as demonstrated in Fig. 6, where E, B, and C are an emitter, a base, and collector terminals, respectively. V_CB_ is fixed as positive bias to always keep the left M1 in a conduction state. While 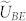 applied on the right M1 can be altered to control an emitter current I_E_. We define 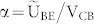. Remarkably, if α > 0, the right M1 can open a large current due to a positive bias being applied. But if α < 0, the conduction of the right M1 is severely suppressed owing to a negative bias effect. According to the nearly linear I-V characteristics in the bias region [0.0, 0.5 V] as displayed in Fig. 4(a) for M1, the currents for a base and collector terminals can be expressed approximately as I_C_ = GV_CB_ and 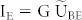, respectively. And also by I_E_ = I_C_ + I_B_. The current amplification coefficient β upon a conventional definition 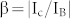 can be given as 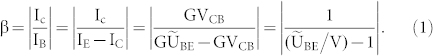
Finally, we obtain 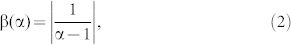
this means that the current amplification coefficient β completely depends on the α value. The β-α relationship based on Eq.(2) is plotted in Fig. 7. When α = 0 or 2.0, we obtain β = 1.0 (no amplification effect). However, the β is enhanced dramatically when α value approaches 1.0, corresponding to a considerable current amplification. This suggests that it is useful for our proposed hybrid structure to build high-performance transistors as well.

## Discussion

By using two kinds of graphene derivatives, ZTG and carbon chains Cn, constructs the all-carbon hybrid structure: Cn/ZTG/Cn, and sandwiching such a structure between two Au electrodes. Our calculations from the first-principles method show that the bonding configurations for its carbon chains would be altered obviously after the nanojunction is formed, which implies that the bonding configurations for carbon chains depend not only on the number of carbon atoms on this chain being odd or even, but also strongly on the nature of their terminal objects. We also find that carbon chains have a very strongly modulating ability to electronic structures and transport of this hybrid structure, especially for its rectifying direction and rectification strength. More interestingly, the high-performance rectifying behaviors similar to a traditional diode, such as a very low threshold voltage, a rather small leakage current, and a wide bias region for rectification, can be predicted. This is mainly because a sp-hybrid carbon chain has a special electronic structure, when its energy level can align with one of the ZTG, the high transmission channel is formed and, simultaneously, just able to asymmetrically shift under biases of different directions, which contributes to a large rectification. In this sense, carbon chains can be viewed as particular spacers between the ZTG and electrodes, which limits the electronic resonant tunneling of the ZTG to a unique and favorable situation in the positive- and negative-bias windows to form a strong rectification. Additionally, a transistor can be built by such hybrid structure, which shows an extremely high current amplification. These results imply that this simple hybrid structure might promise importantly potential applications for developing nano-scale integrated circuits.

## Methods

In our studies, geometric optimizations of the device region and calculations of electronic structure and transport properties are performed by using the density function theory (DFT) combined with the non-equilibrium Green's function (NEGF) method^38^,^39^,^40^. We employ Troullier-Martins norm-conserving pseudopotentials to represent the atom core and linear combinations of local atomic orbitals to expand the valence states of electrons. The Perdew-Burke-Ernzerhof (PBE) formulation of the generalized gradient approximation(GGA) is used as the exchange-correlation functional. Single-zeta plus polarization (SZP) basis set for Au and H atoms and double-zeta plus polarization (DZP) basis set for C and S atoms are adopted. The k-point sampling is 1, 1, and 500 in the x, y, z direction, respectively, where the z is the electronic transport one, and the cutoff energy is set to 150 Ry. Open boundary conditions are used to describe the electronic and the transport properties of nanojunctions. Each nanojunction composes of the left electrode, scattering region (the device region), and right electrode. The scattering region contains molecular core and five layers of Au surfaces on its each side. Its geometries are optimized until all residual forces on each atom are smaller than 0.05 eV/Å.

## Author Contributions

Z.Z. and J.Z. performed the device design and theoretical analysis, G.K., J.L., Z.F. and X.D. calculated geometrical properties, electronic structures, transmission spectra, and the I-V characteristics, and G.T. studied the current amplification. All the authors discussed the results and wrote the manuscript.

## Figures and Tables

**Table 1:** The spatial distribution of molecular states for all models at zero bias

Model	HOMO − 2	HOMO − 1	HOMO	LUMO	LUMO + 1
M1	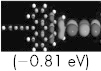	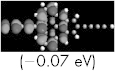	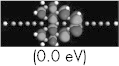	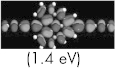	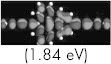
M2	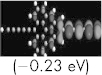	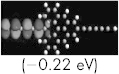	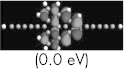	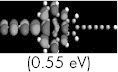	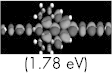
M3	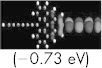	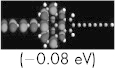	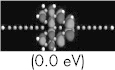	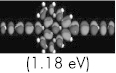	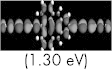
M4